# The impact of early hypoglycemia and blood glucose variability on outcome in critical illness

**DOI:** 10.1186/cc7921

**Published:** 2009-06-17

**Authors:** Sean M Bagshaw, Rinaldo Bellomo, Michael J Jacka, Moritoki Egi, Graeme K Hart, Carol George

**Affiliations:** 1 Division of Critical Care Medicine, University of Alberta Hospital, University of Alberta, 8440-112 ST NW, Edmonton, Alberta, Canada, T6G2B7; 2 Department of Epidemiology and Preventive Medicine, Monash University, Alfred Hospital, Melbourne, Victoria 3004, Australia; 3 Faculty of Medicine, University of Melbourne, 766 Elizabeth Street, Melbourne, Victoria 3010, Australia; 4 Department of Anesthesiology and Pain Medicine, University of Alberta Hospital, University of Alberta, 8440-112 ST NW, Edmonton, Alberta, T6G 2B7 Canada; 5 Department of Anesthesiology and Resuscitology, Okayama University Hospital, 2-5-1 Shikata-cho, Okayama, 700-8558 Japan; 6 Australia New Zealand Intensive Care Society (ANZICS) Adult Patient Database (APD), 10 Ievers Terrace, Carlton, Victoria 3053, Australia

## Abstract

**Introduction:**

In critical illness, the association of hypoglycemia, blood glucose (BG) variability and outcome are not well understood. We describe the incidence, clinical factors and outcomes associated with an early hypoglycemia and BG variability in critically ill patients.

**Methods:**

Retrospective interrogation of prospectively collected data from the Australia New Zealand Intensive Care Society Adult Patient Database on 66184 adult admissions to 24 intensive care units (ICUs) from 1 January 2000 to 31 December 2005. Primary exposure was hypoglycemia (BG < 4.5 mmol/L) and BG variability (BG < 4.5 and ≥ 12.0 mmol/L) within 24 hours of admission. Primary outcome was all-cause mortality.

**Results:**

The cumulative incidence of hypoglycemia and BG variability were 13.8% (95% confidence interval (CI) = 13.5 to 14.0; n = 9122) and 2.9% (95%CI = 2.8 to 3.0, n = 1913), respectively. Several clinical factors were associated with both hypoglycemia and BG variability including: co-morbid disease (*P *< 0.001), non-elective admissions (*P *< 0.001), higher illness severity (*P *< 0.001), and primary septic diagnosis (*P *< 0.001). Hypoglycemia was associated with greater odds of adjusted ICU (odds ratio (OR) = 1.41, 95% CI = 1.31 to 1.54) and hospital death (OR = 1.36, 95% CI = 1.27 to 1.46). Hypoglycemia severity was associated with 'dose-response' increases in mortality. BG variability was associated with greater odds of adjusted ICU (1.5, 95% CI = 1.4 to 1.6) and hospital (1.4, 95% CI = 1.3 to 1.5) mortality, when compared with either hypoglycemia only or neither.

**Conclusions:**

In critically ill patients, both early hypoglycemia and early variability in BG are relatively common, and independently portend an increased risk for mortality.

## Introduction

Elevated blood glucose (BG) levels and stress-hyperglycemia have been identified as modifiable risk factors for adverse outcomes in critically ill patients. [1]. Randomized trials of intensive monitoring and insulin therapy (IIT) in critically ill patients have been performed showing improvements in morbidity and mortality with tight glycemic control (TGC) [2-6]. Data from selected trials suggest that, for TGC to exert its clinical benefit, BG values must be maintained in the range of 4.4 to 6.1 mmol/L [4,5]. Based on this evidence, use of IIT to achieve TGC has been widely advocated to improve outcomes for critically ill patients [7,8]. Recently, however, the findings of the multi-center multi-national NICE-SUGAR (Normglycemia in Intensive Care Evaluation – Survival Using Glucose Algorithm Regulation) randomized trial, comparing IIT with less-intensive insulin therapy in 6104 critically ill patients, have suggested the use of IIT is associated with a higher 90-day mortality (27.5% vs. 24.9%; odds ratio (OR) = 1.14, 95% confidence interval (CI) = 1.02 to 1.28, *P *= 0.02). [9].

Accordingly, the issue of TGC remains controversial [10-17]. Concerns have arisen that TGC may be associated with unacceptably high rates of hypoglycemia [18]. In the two TGC trials in surgical and medical patients from the University of Leuven, hypoglycemia (BG < 2.2 mmol/L) occurred in 5.1% and 18.7% of patients, respectively [4,5]. Recently, the VISEP (Volume Substitution and Insulin Therapy in Severe Sepsis) trial, a multi-center randomized trial comparing IIT with conventional therapy in critically ill septic patients, was terminated early due to a lack of evidence of survival benefit with IIT and a significantly higher incidence of hypoglycemia in those allocated to IIT (17.0% vs. 4.1%, *P *< 0.001). [19]. Likewise, in the NICE-SUGAR trial, IIT was associated with greater hypoglycemia (6.8% vs. 0.5%, *P *< 0.001) compared with conventional glycemic control. Hypoglycemia in critically ill patients may have unrecognized clinical importance. Observational data have indicated that even a single episode of hypoglycemia may be associated with worse clinical outcomes. [20]. Also, variability in glycemic control has increasingly been recognized as having a potentially important association with clinical outcome [21-24].

Accordingly, we searched the Australian and New Zealand Intensive Care Society (ANZICS) Clinical Outcomes and Resource Evaluation (CORE) Adult Patient Database (APD) to obtain information on BG measures within 24 hours of intensive care unit (ICU) admission in a large cohort of ICU patients from 24 ICUs over a six-year period [25]. Our objectives were to: describe the incidence of and clinical factors associated with an early episode of hypoglycemia and BG variability (within 24 hours of ICU admission) in critically ill patients; evaluate any association between early hypoglycemia, BG variability, and mortality; and evaluate any association between severity of early hypoglycemia and mortality.

## Materials and methods

### Study population

This was a retrospective analysis of prospectively collected data. We searched the ANZICS CORE APD for all adult (age ≥ 18 years) ICU admissions from 1 January 2000 to 31 December 2005. Patients were excluded if data on either BG or outcome were unavailable (7.5%, n = 5329). The ANZICS CORE APD captures clinical, physiologic, and laboratory data for the initial 24 hours of ICU admission for those with a duration of stay of 24 hours or longer, along with outcome data and vital status at hospital discharge. This comprised data from 24 ICUs (10 tertiary referral, 7 metropolitan, 5 regional/rural, and 2 private hospitals) that contributed data over these consecutive years.

Access to the data was granted by the ANZICS CORE Management Committee in accordance with standing protocols. Local hospital Research Ethics Board approval was waived. By government legislation, investigators are allowed to use de-identified data from the APD for the purpose of epidemiologic research so long as it is approved by the CORE Management Committee. Such data are collected and transferred from hospitals to the database under government support and funding with each hospital allowing such transfer and subsequent data use as necessary.

### Blood glucose measures

The APD prospectively captures data on highest (BG_HIGH_) and lowest (BG_LOW_) BG concentrations within the initial 24 hours of ICU admission. All BG values entered into the database are, for the vast majority of patients, obtained via blood gas analyzers and reflect whole BG values. For each patient, we extracted data on the BG_HIGH _and BG_LOW_. We calculated the average BG concentration (BG_AVE_) for the first 24 hours as the mean of the BG_HIGH _and BG_LOW_. This BG_AVE _may not be representative of the true BG_AVE _for those patients having had multiple BG measurements in the first 24 hours.

An episode of hypoglycemia was defined by a documented BG of less than 4.5 mmol/L. Hypoglycemia was further stratified into six mutually exclusive groups of severity by dividing BG_LOW _into the following categories: less than 2.0 mmol/L; 2.0 to 2.4 mmol/L; 2.5 to 2.9 mmol/L; 3.0 to 3.4 mmol/L; 3.5 to 3.9 mmol/L; 4.0 to 4.4 mmol/L; and 4.5 mmol/L or higher. An elevated BG was defined by a documented BG of more than 6.1 mmol/L. We defined early BG variability as any patient who had both an episode of hypoglycemia (BG < 4.5 mmol/L) and hyperglycemia (BG ≥ 12.0 mmol/L) within 24 hours of ICU admission [23].

Data from a large multi-center survey of practice found less than 10% of all ICUs in ANZICS had adopted TGC protocols with IIT following the reporting of the trial by van den Berghe and colleagues [26]. Accordingly, the hypoglycemia occurring in the majority of patients in this study was more likely to be related to primary diagnosis or illness severity rather than IIT.

### Data collection

Standard demographic, clinical, and physiologic data were retrieved. Demographic information included age, sex, and dates and sources of admissions. Clinical data encompassed primary diagnosis, surgical status, co-morbidities, need for mechanical ventilation, and evidence of acute kidney injury (AKI), defined by the RIFLE classification scheme. [27]. Physiologic data included Glasgow Coma Scale (GCS), vital signs, and urine output. Laboratory data included routine hematology and blood chemistry. Severity of illness was assessed using the Acute Physiology and Chronic Health Evaluation (APACHE) II and III score. The operational definitions for pre-existing co-morbidities and primary diagnostic categories are shown in Additional data file 1.

### Outcomes

Outcomes extracted included ICU and hospital mortality. If patients were readmitted to ICU prior to hospital discharge, subsequent ICU admissions were not included in the analysis of mortality.

### Statistical analysis

The cumulative incidence of early hypoglycemia was calculated by dividing the total number of patients with a documented BG less than 4.5 mmol/L by the number of ICU admissions over the five-year study, and is expressed as a proportion (%) with 95% CI. This was similarly performed for BG variability.

We used descriptive statistics to compare the demographic characteristics, clinical factors, and crude outcomes among patients with and without an episode of hypoglycemia. Normally or near normally distributed variables are reported as means with standard deviations (SD) and compared by Student's t-tests. Non-normally distributed continuous data are reported as medians with inter-quartile ranges (IQR) and compared by Mann Whitney U tests. Differences in proportions among categorical data were assessed using Fisher's exact tests for pair-wise comparisons and chi-squared tests for multiple groups.

The primary outcomes for this study were ICU and hospital mortality. We evaluated the association of both a discrete episode of hypoglycemia, the severity of hypoglycemia, and BG variability on ICU and hospital mortality by multi-variable logistic regression analysis for the entire cohort, and for two *a priori *selected subgroups in those with hypoglycemia: those with a primary septic diagnosis, and mechanically ventilated surgical patients. Covariates were selected for inclusion in the models and included age, sex, co-morbidity, non age-related APACHE II score (subtraction of age-related points from full APACHE II score). [28], surgical status, primary diagnosis, need for mechanical ventilation, AKI, and hospital site. For each model, calibration and discrimination were assessed by the goodness-of-fit test and area under the receiver operator characteristic curve (AuROC), respectively. Data are presented as crude and adjusted OR with 95% CI. In the event of missing data values, data were not replaced or estimated. Analyzes were performed with the use of Intercooled Stata Release 10 (Stata Corp, College Station, TX, USA). Two-sided *P *< 0.05, unadjusted for multiple testing, were considered to indicate statistical significance for all comparisons.

## Results

During the six-year study period, 71,513 patients were admitted to the 24 study ICUs for 24 hours or longer. Of these, 66,184 (92.5%) had complete data for both BG values and clinical outcomes. There were 132,368 BG values in the 66,184 ICU patients. The mean (SD) BG_AVE_, BG_HIGH_, and BG_LOW _were 8.7 (4.6) mmol/L, 10.5 (6.0) mmol/L, and 6.9 (4.1) mmol/L, respectively.

### Hypoglycemia

The cumulative incidence of early hypoglycemia during the six-year study was 13.8% (95% CI = 13.5 to 14.0; n = 9122). The clinical characteristics, acute physiology, and crude outcomes of patients with hypoglycemia are shown in Tables 1 to 3. In 2.1% (n = 1409) of the cohort (18.3% of those with documented hypoglycemia), two episodes of hypoglycemia occurred within the first 24 hours.

**Table 1:** Summary of clinical characteristics and outcomes stratified by hypoglycemia and blood glucose variability

**Characteristic/outcome**	**Total** **(n = 66,184)**	**Hypoglycemic episode only** **(n = 7209)**	**Blood glucose variability** **(n = 1913)**	**Neither** **(n = 57,969)**	***P *value**
**Age (mean [SD]) (years)**	61.1 (18)	59.2 (20)	62.6 (17)	61.4 (18)	<0.001
**Male sex (%)**	59.0	53.4	53.2	59.8	<0.001
**Co-morbid disease (%)**	24.2	25.7	29.8	23.8	<0.001
**Cardiovascular**	10.7	10.4	15.5	10.6	0.016
**Respiratory**	7.8	7.7	9.2	7.8	0.08
**Immunocompromised**	5.1	6.2	6.2	4.9	<0.001
**End-stage kidney disease**	3.6	6.6	7.4	3.1	<0.001
**Metastatic cancer**	2.8	2.7	1.8	2.8	0.03
**Liver disease**	2.5	4.2	3.1	2.3	<0.001
**Hematologic malignancy**	1.5	1.7	1.4	1.5	0.41
**Non-elective admission (%)**	65.9	76.9	79.8	64.1	<0.001
**Surgical admission (%)**	45.7	34.4	27.7	47.6	<0.001
**Cardiovascular (%)**	33.8	30.4	37.3	34.1	<0.001
**Trauma (%)**	9.3	7.4	4.3	9.7	<0.001
**Primary diagnosis (%)**					
**Sepsis/septic shock**	23.0	30.4	26.6	21.9	<0.001
**Respiratory**	13.4	10.0	13.9	13.8	<0.001
**Gastrointestinal (other)**	10.5	10.4	8.1	10.6	0.025
**Cardiac**	10.2	9.1	19.5	10.0	<0.001
**Neurologic**	9.8	7.3	6.3	10.2	<0.001
**Hepatic**	6.3	7.6	5.2	6.2	<0.001
**Metabolic/poisoning**	6.8	11.6	11.2	6.1	<0.001
**Gastrointestinal Bleeding**	3.2	2.8	3.0	3.2	0.09

SD = standard deviation.

**Table 2:** Summary of acute physiology stratified by hypoglycemia and blood glucose variability

**Characteristic**	**Total** **(n = 66,184)**	**Hypoglycemic episode only** **(n = 7209)**	**Blood glucose variability** **(n = 1913)**	**Neither** **(n = 57,969)**	***P *value**
**Illness severity scores:**					
**APACHE II [mean (SD)]**	17.0 (8.3)	18.9 (9.4)	22.1 (9.7)	16.2 (8.0)	<0.001
**APACHE III [mean (SD)]**	56.7 (29.8)	65.0 (36.2)	78.1 (35.3)	55.0 (28.2)	<0.001
**Mechanical Ventilation (%)**	45.6	49.6	55.4	44.8	<0.001
**pH [mean (SD)]**	7.32 (0.1)	7.30 (0.14)	7.26 (0.16)	7.33 (0.12)	<0.001
**Creatinine (umol/L) [median (IQR)]**	90 (70 to 136)	100 (68 to 190)	123 (80 to 225)	90 (70 to 130)	0.001
**Urea (mmol/L) [median (IQR)]**	6.7 (4.6 to 11.2)	7.4 (4.3 to 14.8)	9.9 (6.0 to 17.7)	6.6 (4.6 to 10.6)	0.001
**Urine (L/24 hr) [median (IQR)]**	1.75 (1.04 to 2.56)	1.60 (0.84 to 2.50)	1.70 (0.85 to 2.63)	1.77 (1.08 to 2.56)	0.001

APACHE = Acute Physiology and Chronic Health Evaluation; IQR = intra-quartile range; SD = standard deviation.

**Table 3:** Summary of crude clinical outcomes stratified by hypoglycemia and blood glucose variability

**Clinical outcome**	**Total** **(n = 66,184)**	**Hypoglycemic episode only** **(n = 7209)**	**Blood glucose variability** **(n = 1913)**	**Neither** **(n = 57,969)**	***P *value**
**ICU length of stay (days) [median (IQR)]**	1.9 (1.0 to 4.4)	2.0 (1.0 to 4.6)	2.7 (1.3 to 5.5)	1.9 (1.0 to 4.3)	0.001
**Hospital length of stay (days) [median (IQR)]**	10.7 (5.9 to 21.0)	10.0 (4.4 to 21.5)	11.4 (4.9 to 24.1)	10.7 (6.0 to 20.9)	0.001
**ICU mortality (%)**	11.1	17.3	22.6	9.8	<0.001
**Hospital mortality (%)**	16.9	24.3	30.7	15.5	<0.001

ICU = intensive care unit; IQR = intra-quartile range.

Potential important clinical variables associated with early hypoglycemia included female sex (OR = 1.25, 95% CI = 1.20 to 1.30) and having any co-morbid illness (OR = 1.13, 95% CI = 1.09 to 1.18), specifically end-stage kidney disease (OR = 1.91, 95% CI = 1.78 to 2.05), liver disease (OR = 1.60, 95% CI = 1.45 to 1.75), and being immune-compromised (OR = 1.22, 95% CI = 1.13 to 1.32). Medical (OR = 1.71, 95% CI = 1.64 to 1.78), non-elective admissions (OR = 1.77, 95% CI = 1.70 to 1.86) and those with higher severity of illness (OR = 1.22 per five-point increase in APACHE II, 95% CI = 1.21 to 1.24) were associated with higher odds of hypoglycemia. Primary admission diagnoses of sepsis (OR = 1.41, 95% CI = 1.36 to 1.47) and metabolic disturbance and/or poisoning (OR = 1.77, 95% CI = 1.67 to 1.88) had the higher odds of early hypoglycemia.

Early hypoglycemia was associated with higher crude ICU (18.7% vs. 9.8%; OR = 2.11, 95% CI = 2.00 to 2.25) and hospital (25.6% vs. 15.5%; OR = 1.87, 95% CI = 1.77 to 1.97) mortality rates (Table 3). This association remained evident in multi-variable analysis for both ICU (OR = 1.41, 95% CI = 1.31 to 1.53) and hospital (OR = 1.35, 95% CI = 1.26 to 1.45) mortality.

Those patients having two episodes of hypoglycemia within 24 hours of ICU admission had significantly higher crude and covariate-adjusted ICU and hospital mortality compared with only one episode or no hypoglycemia, respectively (Table 4).

**Table 4:** Summary of crude and adjusted ICU and hospital mortality stratified by occurrence of hypoglycemia, and by blood glucose variability, hypoglycemia or neither

**Blood glucose**	**Incidence** **(%)**	**ICU mortality** **OR (95% CI)**		**Hospital mortality** **OR (95% CI)**	
		
		**Crude**	**Adjusted^‡^**	**Crude**	**Adjusted^§^**
**Early hypoglycemia**					
Two episodes	1409 (2.1)	3.3 (2.9 to 3.7)	2.4 (2.0 to 2.8)	2.7 (2.4 to 3.0)	2.2 (1.9 to 2.5)
One episode only	7713 (11.7)	1.9 (1.8 to 2.1)	1.3 (1.2 to 1.4)	1.7 (1.6 to 1.8)	1.2 (1.1 to 1.3)
No hypoglycemia^¶^	57062 (86.2)	1.0	1.0	1.0	1.0

**BG variability**					
BG variability	1913 (2.9)	2.7 (2.4 to 3.0)	1.5 (1.4 to 1.6)^†^	2.4 (2.1 to 2.6)	1.4 (1.3 to 1.5)^□^
Hypoglycemia	7209 (10.97)	2.0 (1.8 to 2.1)	1.2 (1.1 to 1.4)^†^	1.7 (1.6 to 1.8)	1.2 (1.0 to 1.4)^□^
Neither^¶^	57062 (86.2)	1.0	1.0^†^	1.0	1.0^□^

APACHE = Acute Physiology and Chronic Health Evaluation; AuROC = area under the receiver operator characteristic curve; BG = blood glucose; CI = confidence interval; ICU = intensive care unit; OR = odds ratio.

¶Reference variable

‡ Goodness-of-fit, *P *= 1.0; AuROC 0.89.

§ Goodness-of-fit, *P *= 1.0; AuROC 0.87.

† Goodness-of-fit, *P *= 1.0; AuROC 0.89.

□ Goodness-of-fit, *P *= 1.0; AuROC 0.87.

§‡□† Covariate adjustment for age, sex, surgical status, primary diagnosis, co-morbid illness, non-age-related APACHE II score, mechanical ventilation, acute kidney injury, year, and hospital site.

**Figure 1:**
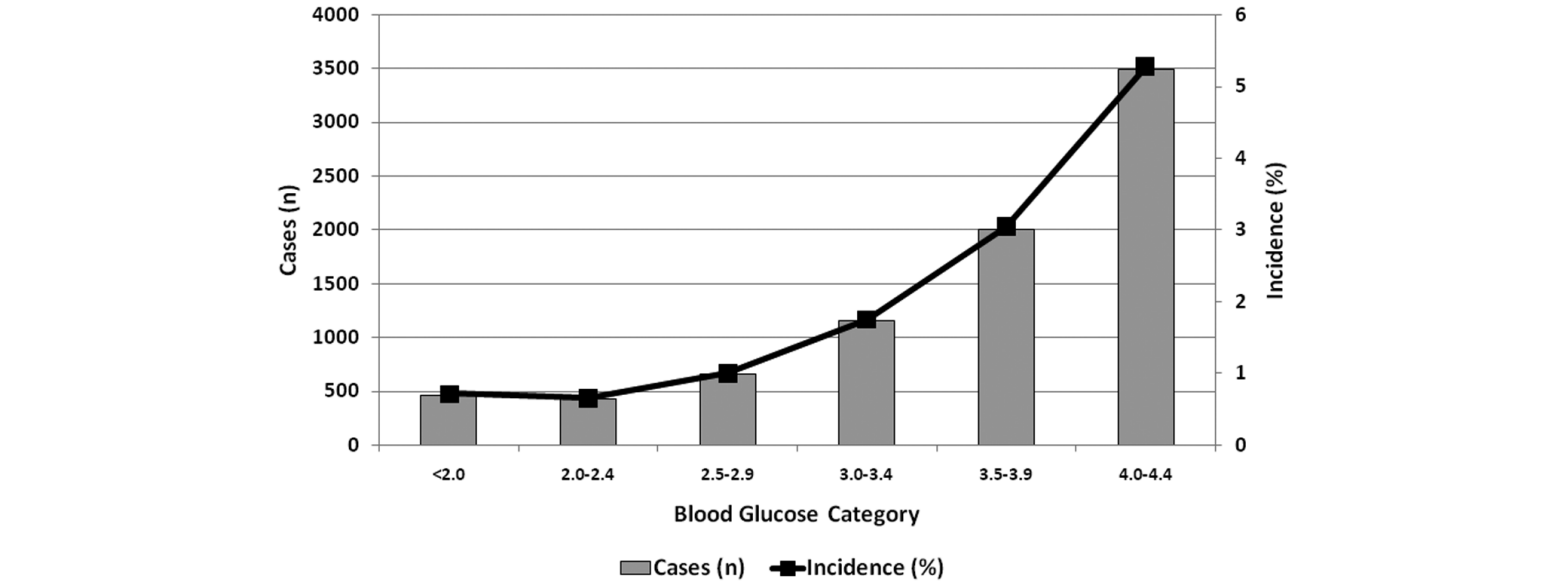
Incidence of hypoglycemia stratified by degree of hypoglycemia within 24 hours of intensive care unit admission.

The occurrence of hypoglycemia stratified by degree of hypoglycemia is shown in Figure 1. Adjusted estimates of ICU and hospital mortality stratified by severity of hypoglycemia are shown in Tables 4 to 6. Increasing severity of hypoglycemia was associated with a 'dose-response' increase in crude and adjusted ICU and hospital mortality. Similar dose-response associations between severity of hypoglycemia and mortality were also apparent in the septic and mechanically ventilated surgical subgroups.

**Table 5:** Adjusted ICU and hospital mortality by severity of hypoglycemia in patients with a primary septic diagnosis

**Blood glucose category** **(mmol/L)**	**ICU mortality** **Adjusted OR^§ ^(95% CI)**	**Hospital mortality** **Adjusted OR^‡ ^(95% CI)**
**<2.0**	4.8 (3.3 to 7.0)	3.8 (2.6 to 5.6)
**2.1–2.4**	2.4 (1.6 to 3.6)	2.1 (1.4 to 3.1)
**2.5–2.9**	1.9 (1.4 to 2.6)	1.7 (1.3 to 2.4)
**3.0–3.4**	2.0 (1.5 to 2.5)	1.8 (1.4 to 2.3)
**3.5–3.9**	1.3 (1.0 to 1.6)	1.3 (1.0 to 1.6)
**4.0–4.4**	1.0 (0.8 to 1.2)	1.0 (0.8 to 1.2)
**≥ 4.5^¶^**	1.0	1.0

APACHE = Acute Physiology and Chronic Health Evaluation; AuROC = area under the receiver operator characteristic curve; CI = confidence interval; ICU = intensive care unit; OR = odds ratio.

¶ Reference variable

§ Goodness-of-fit, *P *= 1.0; AuROC 0.85.

‡ Goodness-of-fit, *P *= 1.0; AuROC 0.82.

§‡ Covariate adjustment for age, sex, surgical status, primary diagnosis, co-morbid illness, non-age-related APACHE II score, mechanical ventilation, acute kidney injury, year, and hospital site.

For conversion of blood glucose from SI to conventional units divide by 0.05551.

**Table 6:** Adjusted ICU and hospital mortality by severity of hypoglycemia in mechanically ventilated surgical admissions [5]

**Blood glucose category** **(mmol/L)**	**ICU mortality** **Adjusted OR^§ ^(95% CI)**	**Hospital mortality** **Adjusted OR^‡ ^(95% CI)**
**<2.0**	6.0 (2.9 to 12.4)	6.4 (3.1 to 13.4)
**2.1–2.4**	1.2 (0.6 to 2.7)	1.2 (0.6 to 2.5)
**2.5–2.9**	1.7 (0.9 to 3.2)	1.9 (1.1 to 3.3)
**3.0–3.4**	1.8 (1.2 to 2.8)	1.7 (1.2 to 2.5)
**3.5–3.9**	1.6 (1.1 to 2.3)	1.5 (1.1 to 2.1)
**4.0–4.4**	1.1 (0.8 to 1.5)	1.1 (0.8 to 1.4)
**≥ 4.5^¶^**	1.0	1.0

APACHE = Acute Physiology and Chronic Health Evaluation; AuROC = area under the receiver operator characteristic curve; CI = confidence interval; ICU = intensive care unit; OR = odds ratio.

¶ Reference variable

§ Goodness-of-fit, *P *= 1.0; AuROC 0.87.

‡ Goodness-of-fit, *P *= 1.0; AuROC 0.84.

§‡ Covariate adjustment for age, sex, surgical status, primary diagnosis, co-morbid illness, non-age-related APACHE II score, mechanical ventilation, acute kidney injury, year, and hospital site.

For conversion of blood glucose from SI to conventional units divide by 0.05551.

### Blood glucose variability

In total, 23% (95% CI = 22.7 to 23.3, n = 15229) of patients had evidence of hyperglycemia (BG ≥ 12 mmol/L) within 24 hours of ICU admission. The cumulative incidence of early BG variability, defined by the presence of both hypoglycemia and hyperglycemia within 24 hours of ICU admission, was 2.9% (95% CI = 2.8 to 3.0, n = 1913; Table 4). When compared with patients with either hypoglycemia only or neither, those experiencing BG variability were older (*P *< 0.001), had a higher burden of co-morbid disease (*P *< 0.001), had higher illness severity (*P *< 0.001), were more likely to be non-elective admissions (*P *< 0.0001), and were significantly more likely to receive mechanical ventilation (*P *< 0.001).

BG variability was also associated with higher crude and covariate-adjusted ICU and hospital mortality when compared with either hypoglycemia only or neither (Table 4).

## Discussion

We conducted a six-year analysis of more than 66,000 individual patient admissions to 24 ICUs across Australia and New Zealand to: describe the incidence of and clinical factors associated with early hypoglycemia and BG variability; evaluate the association between early hypoglycemia, BG variability and mortality; and explore the association between severity of early hypoglycemia and mortality.

We determined that early hypoglycemia (BG < 4.5 mmol/L) is common, occurring in 13.8% of patients within the first day of ICU admission alone (with 18.3% of these patients having two episodes). This is the largest observational study to provide an estimate of the incidence of early hypoglycemia in a general ICU population that does not incorporate a protocol-driven approach for maintaining TCG with IIT. In contrast, in a two-year survey of a single center where TGC by IIT was routinely applied, Vriesendorp and colleagues reported that 6.9% of ICU admissions experienced an episode of severe hypoglycemia (BG < 2.5 mol/L), with 33% of these patients having more than one episode. [29]. In our study, severe hypoglycemia (BG < 2.5 mmol/L) occurred in only 1.4% of patients within 24 hours of ICU admission. This observation is similar to the occurrence of severe hypoglycemia in critically ill patients allocated to the standard/control groups in several IIT trials. [2,4,5,9,19,30]. Conversely, in those allocated to IIT, where routine and strict monitoring was performed, severe hypoglycemia was surprisingly common, occurring in 5 to 19% of patients [2,4,5,9]. Moreover, the high incidence of severe hypoglycemia (8.6 to 12.1%) in patients receiving IIT has also justified the premature termination of two large multi-center randomized trials of TGC in critically ill patients [19,30]. These data suggest the occurrence of hypoglycemia is far more common than appreciated.

We found that several clinical factors were associated with a higher occurrence of early hypoglycemia, suggesting selected patients are at higher risk and may be identifiable. These factors included female sex, pre-morbid end-stage kidney disease, liver disease, being immune-compromised, medical or non-elective admissions, primary diagnosis of sepsis or metabolic/poisoning admission diagnoses, and greater acute severity of illness. Additional factors predisposing to hypoglycemia have been identified that are more likely to be modifiable including adjustments to nutritional support without concomitant adjustment to insulin administration, use of vasoactive medications, and use of continuous renal replacement therapy. [31]. Observational data and findings from randomized trials have shown that TGC with insulin therapy also represents an independent risk factor for hypoglycemia [1,30,31].

Early hypoglycemia in our study was associated with significantly higher ICU and hospital mortality rates, even after adjustment for available confounding factors. Moreover, our findings are further supported by evidence of a dose-response gradient between the severity of hypoglycemia and mortality, along with higher mortality associated with repeated episodes of hypoglycemia. Although numerous studies have concluded that TGC can positively impact the clinical outcomes in ICU patients [1,3,5,6,32,33], the apparent benefit of narrowly regulated glycemic control and IIT may come at the expense of increased rates of hypoglycemia. [34,35]. Data from a single small observational study have suggested no association between severe hypoglycemia and short-term mortality. [29]. However, Brunkhorst and colleagues [19] found that that severe hypoglycemia was independently associated with a higher risk of death (hazard ratio = 3.31, 95% CI = 2.23 to 4.90) with greater duration of stay in hospital [36]. This observation is more consistent with our data, suggesting that any hypoglycemic event may portend an increase in mortality risk. Importantly, despite data to suggest the duration of hypoglycemic episodes are short (largely due to intensive monitoring). [1,30,37], recognition may be delayed and critically ill patients may exhibit impaired counter-regulatory responses, further contributing to poor clinical outcome.

Since publication of the two University of Leuven IIT trials [4,5], several additional randomized trials conducted across a range of critically ill populations have failed to show a benefit in survival and an increased risk of hypoglycemia with IIT compared with conventional therapy. [19,30,38-40]. These data have recently been summarized in a systematic review [18]. Moreover, the NICE-SUGAR trial has found TGC with IIT was associated with an increased risk of death at 90 days. [9]. This recurrent observation raises important questions about what the optimal and safest target for BG control in critically ill patients should be to both optimize clinical outcomes but also prevent the adverse consequences of hypoglycemia, in particular for those with identifiable risks for hypoglycemia. Although our study cannot directly evaluate the impact of TGC with IIT on risk of hypoglycemia or BG variability, we believe this is a critical issue to understand. Moreover, we would suggest that risk modification by TGC may need to be more context specific and that not all critically ill patients may realize the perceived benefits from TGC.

Although avoidance of overt (and sustained) hyperglycemia may have recognized importance for improving clinical outcomes in critically ill patients. [4,5,41], wide variability in glycemic control is increasingly recognized as an important aspect of BG control and has been associated with significantly higher mortality in several observational studies. [21-24,42,43]. We found early variability in BG values occurred in 2.9% of the cohort during the study period. Moreover, those experiencing BG variability showed important differences in several clinical characteristics when compared with those having either hypoglycemia only or neither. For example, these patients were generally older, had higher burden of co-morbid disease, in particular end-stage kidney disease and cardiovascular disease, and had higher illness severity and received greater treatment intensity. More importantly, variability in BG was associated with higher adjusted ICU and hospital mortality when compared with critically ill patients experiencing either hypoglycemia only or neither. In a retrospective analysis of 168,337 BG measurements performed in a cohort of 7049 critically ill patients, Egi and colleagues found variability in BG values were independently associated with increased ICU and hospital death and prolonged duration of ICU stay. [22]. Moreover, this study found BG variability was a more powerful predictor of outcome than average BG values. Similarly, in a prospective observational study of 191 critical ill patients with sepsis receiving IIT, high BG variability (measured by SD of mean BG values) was associated with higher odds of death in multivariable analysis. [43]. In a retrospective analysis of a large cohort of consecutively admitted critically ill patients, Krinsley [24] found the association between BG variability and mortality was strongest for BG in the normal range. In these patients, mortality for those with high BG variability (fourth quartile) was five-fold greater when compared with those with low BG variability (first quartile). Our data would appear to support and extend the findings of these prior investigations by showing that early variability in BG control may negatively impact outcome.

We recognize that there are important limitations to our study that merit discussion. First, our study is observational, not randomized, and is therefore potentially susceptible to bias. Second, we only have available BG values during the first 24 hours of ICU admission. Third, the APD does not capture data on additional factors that may have relevance and, therefore we are unable to comment on whether these modified the risk of early hypoglycemia or BG variability (i.e. early nutritional support, dextrose administration, oral hypoglycemic medications, insulin therapy, or concomitant corticosteroid therapy) and on how, if at all, the hypoglycemia was treated. Thus, we are unable to discriminate early hypoglycemia attributable to the primary diagnosis and illness severity rather than TGC with IIT. It is likely the majority of hypoglycemic episodes in this study were attributable to the primary underlying diagnosis/illness severity rather than IIT. Fourth, we believe that the clinical outcomes associated with BG control in critically ill patients may be modified by pre-existing diabetes [37]; however, we were unable to identify the diabetes subgroup for this study. We recognize this may have been relevant for those patients with BG variability. Fifth, due to our large database many comparisons between groups, in particular for physiologic and laboratory data, achieved statistical significance; however, in many instances these differences have questionable or no clinical relevance (Table 1). Finally, we were unable to evaluate secondary outcome measures (i.e. renal replacement therapy, critical illness neuromuscular complications, nosocomial infections) or data on the potential long-term sequelae of early hypoglycemia or BG variability on outcomes (i.e. cognitive function, survival). We recognize these clinical outcomes have relevance [44,45]. However, we believe our study is strengthened by the large cohort and by the observation of a dose-response gradient and consistency between severity of early hypoglycemia and mortality across several *a priori *planned subgroups.

## Conclusions

In critically ill patients, both early hypoglycemia and BG variability are common, and portend an increased risk of mortality. These observations imply early hypoglycemia and BG variability have clinical relevance and need further evaluation in the context of protocol-driven tight-glycemic control.

## Key messages

• Early hypoglycemia is very common, occurring in an estimated 14% of critically ill patients.

• Several factors were associated with higher risk for early hypoglycemia including: female sex, pre-morbid end-stage kidney disease, liver disease, immune-compromise, medical or non-elective admissions, sepsis or metabolic/poisoning, and greater acute severity of illness. These patients are potentially identifiable factors.

• Early hypoglycemia was associated with clinically relevant increases in ICU and hospital mortality rates, even after adjustment for available confounding factors.

• Early BG variability was relatively common, occurring in 2.8% of all patients admitted to ICU during the study period, and was associated with higher adjusted mortality when compared with patients with either hypoglycemia alone or neither.

• These findings, although limited, imply early hypoglycemia and BG variability have clinical relevance and need further evaluation in the context of protocol-driven tight-glycemic control.

## Abbreviations

AKI: acute kidney injury; ANZICS: Australian and New Zealand Intensive Care Society; APACHE: Acute Physiology and Chronic Health Evaluation; APD: Adult Patient Database; AuROC: area under the receiver operator characteristic curve; BG: blood glucose; CI: confidence interval; CORE: Clinical Outcomes and Resource Evaluation; GCS: Glasgow Coma Scale; IIT: intensive insulin therapy; ICU: intensive care unit; IQR: intra-quartile range; OR: odds ratio; SD: standard deviation; TGC: tight glycemic control.

## Competing interests

The authors declare that they have no competing interests.

## Authors' contributions

SMB and RB were responsible for study conception and design. CG and GKH were responsible for acquisition of data. SMB and RB analyzed and interpreted the data. SMB drafted the manuscript. SMB, MJJ, ME, GKH, CG, and RB critically revised the manuscript.

## Supplementary Material

Additional file 1 A Word file containing the operational definitions for pre-existing co-morbidities and primary diagnostic categories. Click here for file
