# Supplementary material for: The impact of early hypoglycemia and blood glucose variability on outcome in critical illness
Source: Crit Care. 2009 Jun 17;13(3):R91. doi: 10.1186/cc7921 (PMC2717463; doi:10.1186/cc7921)
Supplement: Additional file 1 — A Word file containing the operational definitions for pre-existing co-morbidities and primary diagnostic categories. [file cc7921-S1.doc]

**Additional File 1:**

Pre-existing co-morbidities were defined by use of the chronic health evaluation for APACHE II, APACHE III and SAPS II systems as outlined in the ANZICS APD data dictionary.

- *Cardiovascular disease* was defined by the presence of New York Heart Association (NYHA) class IV symptoms.
- *Respiratory disease* was defined by the presence of chronic restrictive, obstructive or vascular respiratory disease causing severe exercise limitations or documented chronic hypoxemia, hypercapnia, secondary polycythemia or pulmonary hypertension.
- *Liver disease* was defined by the presence of biopsy proven cirrhosis or documented evidence of portal hypertension (i.e. variceal bleeding, encephalopathy).
- *Metastatic cancer* was defined by the presence of any documented metastatic solid organ tumour.
- *Leukemia or lymphoma* was defined by prior documented presence of any form of hematologic malignancy.
- *Immunocompromised* was defined by the presence of advanced disease sufficient to suppress resistance to infection (i.e. malignancy, AIDS) or therapy that suppresses resistance to infection (i.e. chemotherapy, steroids).

Several primary admission diagnostic categories.

- *Sepsis/septic shock* encompassed admissions for primarily sepsis-related diagnoses and included sepsis associated with pneumonia, gastrointestinal disease, urinary tract infections, central nervous system infections, soft tissue infections, and the unique ANZICS APD additions of sepsis with shock of undetermined source.
- *Cardiac diagnosis* encompassed non-surgical admissions with cardiogenic shock, cardiac arrest, congestive heart failure, and acute myocardial infarction.
- *Cardiac surgical* admission was any admission to ICU following any cardiac surgery.
- *Respiratory diagnosis* encompassed primary respiratory arrests, aspiration syndrome, non-cardiogenic pulmonary edema, exacerbations of chronic obstructive pulmonary disease or asthma, and pulmonary embolism.
- *Hepatic diagnosis* included admission with hepatic failure or liver transplant.
- *Gastrointestinal hemorrhage* included bleeding due to peptic ulcers, diverticulosis, and varices. All other non-surgical gastrointestinal diagnoses were categorized as other.
- *Metabolic/poisoning diagnosis* incorporated non-operative causes of metabolic coma, diabetic ketoacidosis, drug overdoses, or other endocrinopathies. *Neurologic diagnosis* incorporated stroke, intracerebral hemorrhage, subarachnoid hemorrhage, epidural hematoma or other neurologic cause for coma.
